# Characteristics and variation of fecal bacterial communities and functions in isolated systolic and diastolic hypertensive patients

**DOI:** 10.1186/s12866-021-02195-1

**Published:** 2021-04-26

**Authors:** Pan Wang, Ying Dong, Kun Zuo, Chunming Han, Jie Jiao, Xinchun Yang, Jing Li

**Affiliations:** Heart Center & Beijing Key Laboratory of Hypertension, Beijing Chaoyang Hospital, Capital Medical University, 8th Gongtinanlu Rd, Chaoyang District, Beijing, 100020 China

**Keywords:** Isolated systolic hypertension, Isolated diastolic hypertension, Gut microbiota, Metagenome

## Abstract

**Background:**

Hypertension (HTN) is one of the major cardiovascular risk factors, which contributes to increasing target organ damages and cardiovascular morbidity and mortality worldwide. Isolated systolic HTN (ISH) and isolated diastolic HTN (IDH) are two important subtypes of HTN. Previous researches have demonstrated the alteration of fecal bacteria in HTN, but not down to these two sub-types. In order to identify whether the composition of bacterial taxa and functional modules shift in ISH and IDH, we performed a metagenomic sequencing analysis of fecal samples from 15 controls, 14 ISH, and 11 IDH.

**Results:**

Compared with control and ISH, IDH patients showed decreased gene number, bacterial richness, and evenness, although the bacterial alterations did not reach statistical significance in the Shannon index. Also, at the genus level, the β-diversity for intestinal flora in IDH was distinguishable from those with ISH. Furthermore, the taxonomic composition of ISH or IDH was different from that of healthy control at genus and species levels. Patients with IDH or ISH were confirmed to be enriched with *Rothia mucilaginosa*, along with reduced *Clostridium sp. ASBs410*. Lastly, the altered KEGG modules were significantly decreased in IDH compared with the control group, such as sodium transport system; while for ISH, functions relevant to biotin biosynthesis were decreased.

**Conclusions:**

Overall, our results showed the disordered fecal bacteria profiles in subjects with ISH and especially IDH, emphasizing the significance of early intervention for IDH.

**Supplementary Information:**

The online version contains supplementary material available at 10.1186/s12866-021-02195-1.

## Introduction

Hypertension (HTN) is one of the major cardiovascular risk factors, which substantially contributed to increasing target organ damage, cardiovascular morbidity and mortality worldwide [1]. Accompanied by increasing urbanization and related changes in lifestyle, rising income, and population aging, the burden of HTN has been on the rise, especially in China [2–4]. A large representative survey recently showed that the prevalence of HTN among adults aged ≥18 years reached up to 23.2% in China, affecting approximately 244.5 million individuals [5]. Accumulating evidence has been indicating that both genetic susceptibility and environmental factors are implicated in the development of HTN [6, 7]. In particular, considering the acknowledgment of the role of fecal bacteria in non-communicable diseases, such as metabolic diseases deepen [8–10], the relationship between fecal bacteria and HTN has also been advanced in recent years.

Changes in the bacterial composition do not necessary indicate a dysbiosis condition, and bacterial community might find new stable configuration that not result in dysbiotic conditions according to the concepts of microbial resilience proposed by investigators in recent years [11]. Resilience, together with resistance and functional redundancy, are the ability for the intestinal microbiota to restore into the original function, and is known as key properties of a robust microbiota [12]. And dysbiosis as a new state, refers to an imbalance in the taxonomic composition of gut microbiota, which occurs when resilience of the community fails, and would lead to various disorders such as chronic inflammatory diseases and metabolic syndrome [11, 12]. Emerging investigations have identified the aberrant fecal bacteria in various animal models [13, 14], and hypertensive patients [15], such as a decreased bacterial diversity, disordered microbial structure and functions. For instance, significant decrease in microbial richness and evenness was detected in hypertensive animals and individuals [13–15]. In addition, compared with the healthy controls, *Prevotella*-dominated gut enterotype, distinct metagenomic composition with reduced bacteria related to healthy status and overgrowth of bacteria such as *Prevotella* and *Klebsiella* in hypertensive populations was reported [15]. Furthermore, elevated blood pressure (BP) was demonstrated to be elicited by transferring intestinal microbiota from hypertensive human donors to germ-free mice. The causal role of fecal bacteria dysbiosis in contributing to HTN was confirmed [15].

Populations suffering HTN display a wide phenotypic variability, and different types of HTN might be attributable to distinct underlying mechanisms. To the best of our knowledge, there are isolated systolic HTN (ISH) and isolated diastolic HTN (IDH) where patients exhibit abnormal elevation in solely systolic BP (SBP) or diastolic BP (DBP). Individuals with these subtypes may be characterized in hemodynamic or structural abnormalities, respectively, during the development of HTN [16]. In detail, ISH has been frequently associated with enhanced stiffness in large artery [17], while IDH is more relevant to increased peripheral vascular resistance [18]. However, the changes in composition and function of gut microbes in human patients suffering ISH or IDH remain unexplored. Therefore, we proposed a hypothesis that the composition of bacterial taxa and functional modules might be altered in ISH or IDH patients. Thus, we herein conducted a shotgun metagenomic analysis of fecal bacteria in ISH and IDH along with healthy controls.

## Materials and methods

### Study cohort

The subjects were enrolled from a previous study [15]. All the individuals in the present study were from a cohort study among employees of the Kailuan Group Corporation. None of the them was under antihypertensive treatment. Patients suffering from cancer, heart failure, renal failure, stroke, and peripheral artery disease were excluded. Furthermore, individuals were also excluded if they had received antibiotics or probiotics within the last 2 months. In the current study, the participants were divided into three groups: 15 healthy controls (SBP ≤125 mmHg, or DBP ≤80 mmHg), 11 patients with IDH (DBP ≥90 mmHg and SBP < 140 mmHg) [19] and 14 patients with ISH (SBP ≥140 mmHg and DBP < 90 mmHg) [19]. Ethics approval was obtained from Kailuan General Hospital, Beijing Chaoyang Hospital. All the participants signed informed consent forms prior to data collection.

### DNA extraction and library preparation

Stool samples were freshly collected in a sterile container from each participant and immediately froze at − 20 °C. Samples were shipped to the laboratory with ice pack and stored at − 80 °C until analysis. On the basis of manufacturer’s recommendations, DNA was extracted using TIANGEN kit from Novogene Bioinformatics Technology Co., Ltd. Qualified DNA was fragmented by ultrasonic processor, and library of approximately 300 bp clone insert sizes was constructed per sample. Paired-end metageomic sequencing was conducted by Illumina platform with read length 150 bp, insert size 300. The reads aligned to the human genome (alignment with Short Oligonucleotide Analysis Package 2 [SOAP2], Version 2.21, parameters: -s 135, −l 30, −v 7, −m 200, −× 400) were removed after quality control, and remaining high-quality reads were used for analysis.

### Bacterial diversity analysis

Rarefaction curves were conducted to evaluate whether the sample size is enough by randomly selecting a certain number of individuals from the samples and counting the number of species represented by these individuals. In addition, to identify whether the sequencing quantity is sufficient, rarefaction curves by gradually expanding the sequencing depth of random sampling was also performed. The α-diversity parameters of Shannon index, Chao richness, and Pielou evenness were computed using the vegan R package (version 3.3.3) at the genus and species levels, differences of α-diversity indices among groups were tested by Kruskal-Wallis test. Also, β-diversity metrics, including nonmetric dimensional scaling (NMDS), principal-component analysis (PCA), principal coordinate analysis (PCoA) and Permutational Multivariate Analysis of Variance (PERMANOVA) were carried out based on abundances of the microbes at genus and species levels. NMDS was calculated by the vegan package, PCA by the FactoMineR package, PCoA and PERMANOVA by the vegan and ape packages in R software (version 3.3.3). Furthermore, as previously described [20, 21], we evaluated differences in the distribution of β-diversity on axes by wilcoxon rank sum test.

### Taxonomic annotation and abundance profiling

DIAMOND (version 0.7.9.58, default parameters except that −k 50 − sensitive −e 0.00001) was used to align the clean reads to the integrated NR database [22]. Only genes with e-values ≤10 × e-value of the top hit mapped reads were retained to distinguish taxonomic groups [15]. The taxonomic level of each gene was generated following the procedure described by Huson et al. [23]. The taxonomic relative abundance profile was calculated by summing the abundance of genes annotated to the same feature.

### Functional analysis

Functional annotation was carried out by DIAMOND (Version 0.7.9.58, default parameters except that −k 50 − sensitive −e 0.00001) against the Kyoto Encyclopedia of Genes and Genomes (KEGG) (Release 73.1, with animal and plant genes removed) database [24]. Each protein was assigned to KEGG modules by the highest scoring annotated hit (s) containing at least one high-scoring segment pair scoring > 60 bits [25].

### Statistical analysis

Quantitative variables were expressed as median and interquartile, and Wilcoxon rank-sum test was used for between-group comparisons. Categorical variables were presented as numbers and compared using Chi-square test. Differential abundance of genera, species, and KEGG modules were determined using the Kruskal-Wallis test, and *P* values were corrected for multiple comparisons using the Benjamini-Hochberg method, shown as *q* values. Correlations between shared differential signatures in ISH and IDH with clinical factors were estimated by Spearmen’s correlation analysis. All statistical analyses were performed by R software (version 3.3.3), and *P* or *q* < 0.05 was regarded as statistical significance.

## Results

### Baseline characteristics of the study cohort

In the current study, 40 participants, including 15 healthy controls, 14 ISH, and 11 IDH, were recruited from the previous study [15]. The clinical characteristics of all participants are shown in Table 1. Briefly, compared to controls, ISH and IDH patients presented with extremely higher SBP and DBP, respectively (*P* values < 0.01). Also, both SBP and DBP levels between ISH and IDH were dramatically different as expected. In addition, there was no significant difference in other clinical characteristics, such as body mass index, uric acid, creatinine, fasting blood glucose, total cholesterol, triglyceride, low-density lipoprotein, and high-density lipoprotein between groups.

**Table 1:** General characteristics of study participants

Characteristics	Nor	ISH	IDH	*P*_1_-Value (Nor vs. ISH)	*P*_2_-Value (Nor vs. IDH)	*P*_3_-Value (ISH vs. IDH)
Number	15	14	11			
Age, years	52 (49–61)	59.5 (49.75–63)	51 (48–57)	0.264	0.735	0.062
Male/female sex	15 (12/3)	14 (14/0)	11 (10/1)	0.125	0.614	0.440
Systolic BP	118 (113–122)	155 (144.5–164.58)	138.33 (120–140)	0	0.001	0
Diastolic BP	75.67 (70–80)	80.33 (79–85.83)	96 (91.33–100)	0.01	0	0
HR	71.5 (66.75–75.5)	74.5 (66–81.25)	71.5 (67.5–75.5)	0.66	0.909	0.769
Body mass index	24.9559 (23.06–27.16)	25.93 (23.18–28.32)	25.39 (24.21–29.40)	0.535	0.642	0.956
Height	169.5 (164.75–174.25)	170 (167.5–172)	173 (167–175)	0.781	0.492	0.332
Weight	69.5 (65–80.5)	73.5 (67.25–85.25)	75 (70–82)	0.38	0.206	0.66
Uric acid	332 (278–372)	335 (307–433.5)	354 (324–421)	0.596	0.253	0.543
Creatinine	70 (65–86)	71 (62.5–99.9)	71 (60–86)	0.444	0.959	0.511
Fasting blood glucose	5.21 (5.05–5.43)	5.53 (5.18–5.97)	5.37 (4.82–5.58)	0.064	0.736	0.18
Total cholesterol	5.09 (4.7–5.74)	5.55 (4.95–6.34)	5.09 (4.95–5.86)	0.176	0.64	0.396
Triglyceride	1.04 (0.95–1.7)	1.28 (0.91–1.68)	1.86 (1.05–3.64)	0.983	0.102	0.09
LDLC	2.68 (2.33–2.89)	2.87 (2.34–3.71)	2.04 (1.78–2.76)	0.198	0.113	0.055
HDLC	1.24 (1.08–1.39)	1.65 (1.2–2.09)	1.18 (0.95–1.44)	0.081	0.363	0.063
Hemoglobin	151 (143–163)	159 (154.75–163.75)	161 (146–165)	0.162	0.392	0.978
Blood platelet	230 (189–302)	221.5 (177.5–236.75)	243 (193–255)	0.176	0.622	0.208
white blood cell	5.5 (5.1–6.8)	5.7 (4.74–7.1)	6.3 (5.8–7.2)	0.484	0.311	0.298

*P*_1_ value: Nor versus ISH. *P*_2_ value: Nor versus IDH, *P*_*3*_ value: ISH versus IDH

Nor: Healthy controls; *ISH* Isolated systolic HTN, *IDH* Isolated diastolic HTN, *HR* Heart rate, *HTN* Hypertension, *BP* Blood pressure, *LDLC* Low-density lipoprotein cholesterol, *HDLC* High-density lipoprotein cholesterol

### Gut microbial diversity of ISH and IDH

**Fig. 1:**
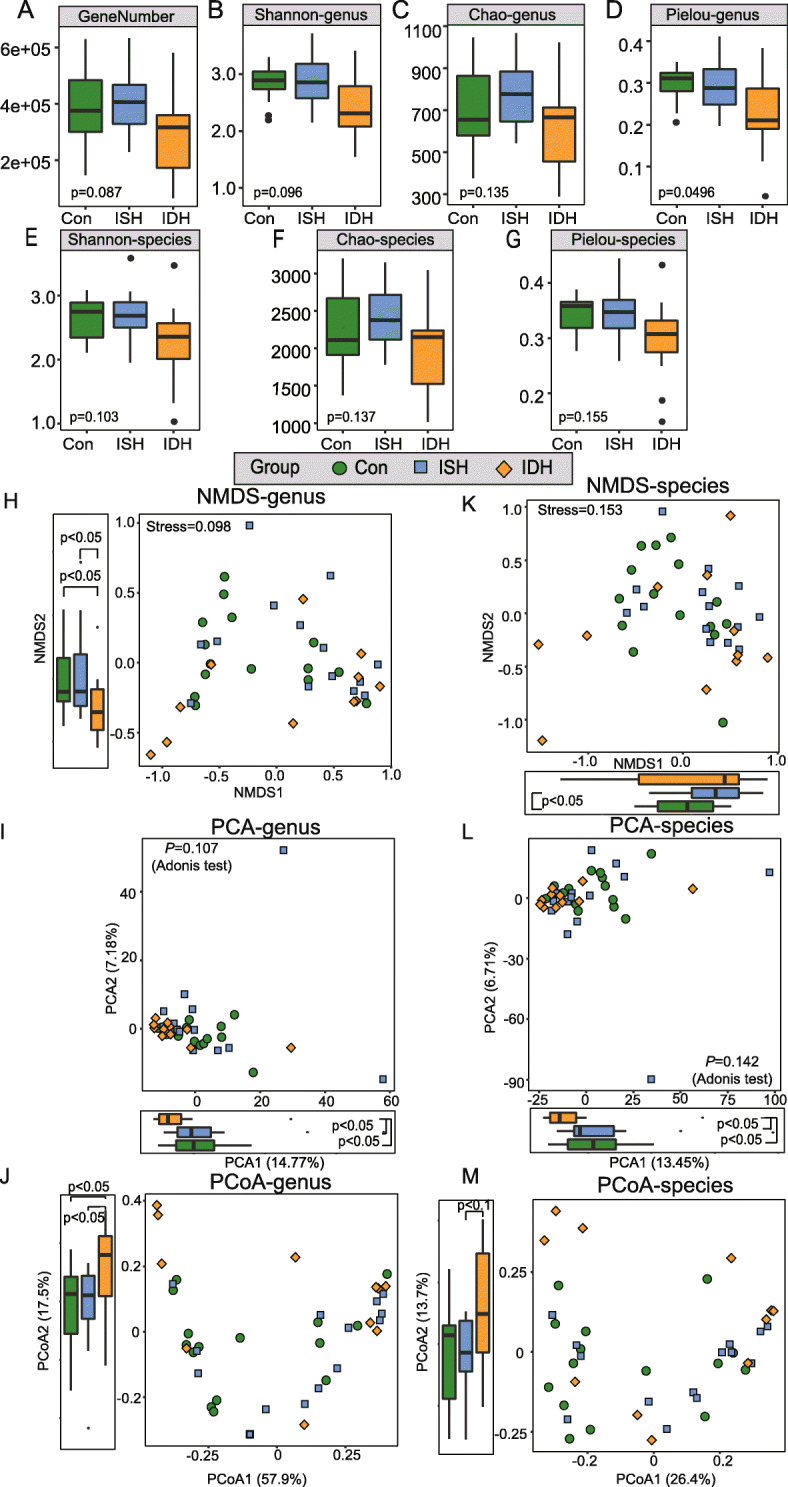
Shifts of intestinal bacteria in α- and β-diversity in patients with IDH and ISH. **a**-**d** Box plots show gene number, Shannon index, Chao richness, and Pielou evenness at each group’s genus level. **e**-**g** Box plots show Shannon index, Chao richness and Pieloue evenness at each group’s species level. Green, con, control, *n* = 15; blue, ISH, *n* = 14; orange, IDH, *n* = 11. **h**-**j** NMDS, PCA, and PCoA plots based on the genera level in groups. Significant differences across groups were established at NMDS2, PCA1, and PCoA2. Circles in green indicate samples from control, squares in blue indicate samples from ISH, rhombus in orange represent individuals from IDH. **k**-**m** Scatter diagram showing NMDS, PCA, and PCoA plots at the species level. Boxes represent the interquartile ranges; the inside lines represent the median; circles represent outliers. For gene number and α-diversity indices, *P* values were obtained by the Kruskal-Wallis test. For β-diversity metrics, *P* values were obtained by PERMANOVA tests; while the differences in the distribution of β-diversity on axes were obtained by wilcoxon rank sum test

A total of 223.03 Gb 125 bp paired-end reads were generated from the raw data (244.67 Gb), with an average of 5.5759 ± 0.6260 (s.d.) million reads per sample (Table S1). The assembled long contigs or scaffolds by high-quality sequencing readings were used for gene prediction, taxonomic classification, and functional annotation. Rarefaction curves were conducted to evaluate whether the sample size is enough by randomly selecting a certain number of individuals from the samples and counting the number of species represented by these individuals. We found the curves tend to be flat, indicating that the sample size is sufficient and reasonable, and only a small number of new species characteristics would be produced by more sampling (Fig. S1A). In addition, to identify whether the sequencing quantity is sufficient, rarefaction curves by gradually expanding the sequencing depth of random sampling was performed. Fig. S1B demonstrated that the curves approached saturation as the sample sequencing depth increases, and thus the amount of sequencing data is sufficient and stable. For the α-diversity, although we did not find significant difference among three groups, participants with IDH had deficient genes and a trend toward lower Shannon index, Chao richness and Pielou evenness compared with healthy controls and patients with ISH at both genus (Fig. 1b-d) and species (Fig. 1e-g) levels.

Furthermore, β-diversity observed in NMDS, PCA, and PCoA plots at genus and species levels suggested that participant grouping had a certain effect on bacteria structure (Fig. 1h-m). No obvious separation was seen in bacterial community by patient categories either at the genus (Fig. 1h-j) or species level (Fig. 1k-m). Yet, the distribution of samples in axis (second NMDS, first PCA and second PCoA at genus level; and first PCA at species level) differed when comparing IDH with control and ISH, suggesting a difference of β-diversity to some extent (β-diversity at genus level: Fig. 1h-j; *P*_NMDS2 (Control vs. IDH)_ = 0.0414, *P*_NMDS2 (ISH vs. IDH)_ = 0.0442; *P*_PCA1 (Control vs. IDH)_ = 0.0204, *P*_PCA1 (ISH vs. IDH)_ = 0.0211; *P*_PCoA2 (Control vs. IDH)_ = 0.0237, *P*_PCoA2 (ISH vs. IDH)_ = 0.0179; β-diversity at species level: Fig. 1l; *P*_PCA1 (Control vs. IDH)_ = 0.0316, *P*_PCA1 (ISH vs. IDH)_ = 0.0333).

In addition, to identify how heterogeneity among samples within the same category as other investigators previously proposed [26, 27], the dispersion of replicates within each group was evaluated for both bacterial diversity and patient phenotypic variability. A similar within β-diversity for bacterial community was detected (Fig. S2A). Instead, individuals in groups vary more in phenotypic variability (Fig. S2B).

### Bacteria profiling and comparison of bacteria composition between groups

**Fig. 2:**
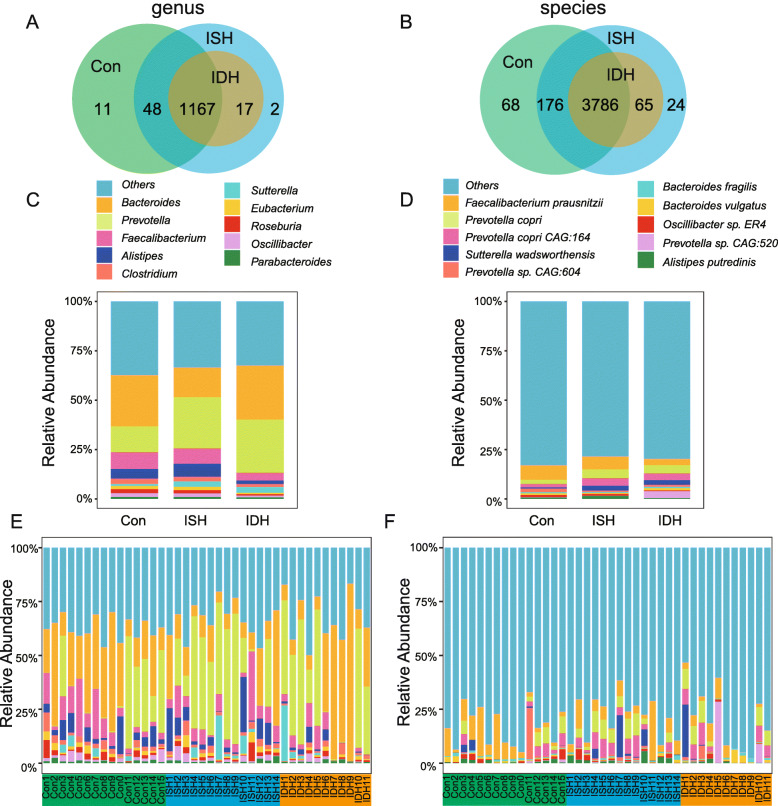
Genera and species annotated in the stool of control, ISH, and IDH. **a, b** Venn diagrams showing the number of genera and species annotated in groups. A total of 1167 genera and 3786 species were shared in control (con), ISH, and IDH groups. **c**, **d** Bar plots showed relative abundance and proportion of the top 10 genera and species in each group. Genera and species are differentiated by color. **e**, **f** Bar plots showed relative abundance and proportion of the top 10 genera and species in each sample. Genera and species are differentiated by color. Others in Fig. S2C and S2D indicates the sum of all the other genera/species expect the top 10 genera/species

Next, taxonomic annotation and abundance profiles of bacteria composition were assessed among groups (Fig. 2). Our results revealed that regardless of the genus (Fig. 2a) or species (Fig. 2b) level, patients with IDH had a lower number of taxa annotated than those with ISH and controls, which is consistent with lower α-diversity obtained in Fig. 1. In addition, a relative abundance of the top ten most abundant genera, including *Bacteroides*, *Prevotella,* and *Faecalibacterium*, and the top 10 predominant species, including *Faecalibacterium prausnitzii*, *Prevotella copri*, *Prevotella copri CAG:164* were assessed in each group (Fig. 2c-d) and in each sample (Fig. 2e-f). The global taxa composition differed across groups. Compared with ISH patients, those with IDH were characterized by a lower proportion of *Alistipes* and *Faecalibacterium* at the genus level and *Faecalibacterium prausnitzii* at the species level.

**Fig. 3:**
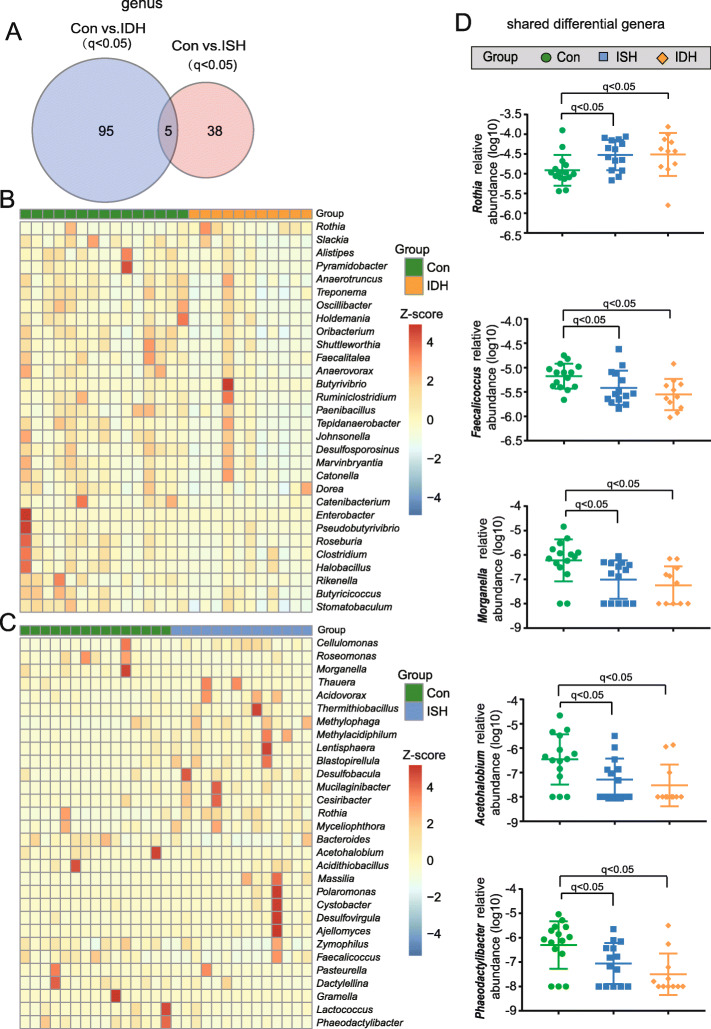
Significantly different Genera between control and ISH, control and IDH, respectively. **a** Venn diagrams demonstrating the genera number were significantly altered when comparing control (con) and IDH, control (con), and ISH. The overlap identified concurrently altered 5 genera; *q*<0.05 (*P* values were corrected using the Benjamini-Hochberg method, shown as *q* values), Wilcoxon rank-sum test. **b** Heat map showing the top 30 of the 100 differential genera between control and IDH. The abundance profiles were expressed by Z scores. The Z score was negative (shown in blue) when the row abundance was lower than the mean and is shown in red when the row abundance was higher than the mean. **c** Heat map for the top 30 of the 43 genera significantly shifted in all individuals with ISH compared to healthy controls. **d** Scatter plots indicating relative abundance of the 5 differential genera shared between control vs. IDH and control vs. ISH. In order to clearly show it, the relative abundance of genera was transformed into log10 values. Green, control; blue, ISH; and orange, IDH. The dots indicate individual values of the participants, and the horizontal lines from bottom to top represent 25th percentiles, medians, and 75th percentiles, respectively

**Fig. 4:**
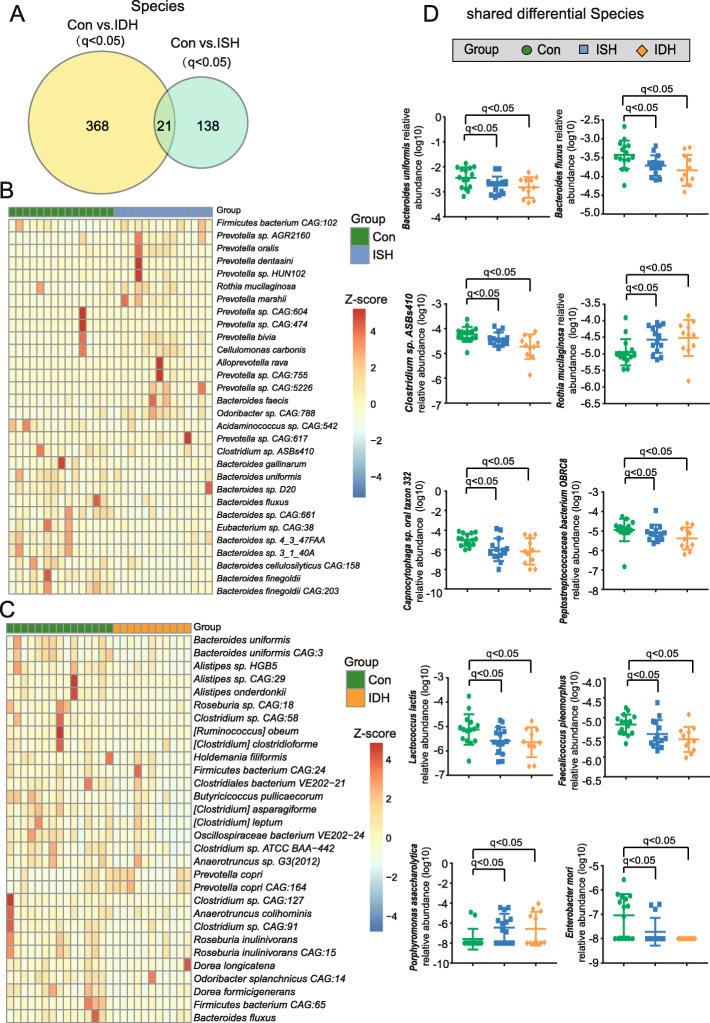
Significantly distinct Species in IDH and ISH as compared with controls. **a** Venn diagrams demonstrating the number of differential species on comparing control (con) and IDH, control, and ISH, respectively. The overlap identified 21 concurrently varied species; *q*<0.05 (*P* values were corrected using the Benjamini-Hochberg method, shown as *q* values), Wilcoxon rank-sum test. **b** Heat map showing the top 30 of the 389 altered species in IDH. The abundance profiles were expressed by Z scores. The Z score was negative (shown in blue) when the row abundance was lower than the mean and is shown in red when the row abundance was higher than the mean. **c** Heat map showing the top 30 of the 159 different species in control and ISH. **d** Scatter plots show the relative abundance of the top 10 species shared between control vs. IDH, and control vs. ISH. In order to show it clearly, the relative abundance of species-level was transformed into log10 values. Green, control; blue, ISH; and orange, IDH. The dots indicate individual values of the participants, and the horizontal lines from bottom to top represent 25th percentiles, medians, and 75th percentiles, respectively

Next, direct comparisons and differential analyses in bacteria abundance among these groups were performed at genus (Fig. 3) and species (Fig. 4) levels so as to identify a number of discriminatory taxa that were differentially abundant. Compared with healthy controls, intestinal bacteria of 100 genera (Fig. 3a) and 389 species (Fig. 4a) significantly differed in abundance from those with IDH. Furthermore, 43 genera (Fig. 3a) and 159 species **(**Fig. 4a) significantly differed between ISH and healthy controls. Of note, 26 shared taxa with 5 genera and 21 species had significantly different abundance in IDH and ISH, which strongly suggested that their modulation was related to both IDH and ISH (Fig. 3a and Fig. 4a). As compared to controls, the top 30 altered genera in IDH and in ISH are shown in Fig. 3b-c, respectively. Likewise, Fig. 4b-c show the top 30 out of the 389 differential species in IDH and from the 159 in ISH, respectively.

Intriguingly, it was worth noting that almost all these bacterial genera and species exhibited a deficiency in IDH, indicating a shift in taxonomic composition of fecal bacteria in patients with IDH. In addition, the relative abundance for the 5 shared genera (Fig. 3)d and top 10 of the 21 species (Fig. 4d) that synchronously shifted in ISH and IDH was further shown, particularly, the bacteria that were more abundant in ISH than controls and that showed higher enrichment in IDH, such as *Rothia* at the genus level and *Rothia mucilaginosa* at a species level. On the other hand, in the IDH group, we also found a much lower abundance for the taxa decreased in ISH, including *Faecalicoccus* and *Acetohalobium* at the genus level and *Clostriduim sp. ASBs410*, *Bacteroides uniformis*, and *Bacteroides fluxus* at the species level. Thus, more disturbed shifts might be present in populations suffering from IDH.

In addition, to further identify which microbes are correlated with which diseases cues or host metabolic change, the phenotypic variability of patients including sex, age, SBP, DBP and other demographic characteristics was correlated with the bacterial taxonomy by Spearmen’s correlation analysis. We found no matter at genus (Fig. S3) or species (Fig. S4) levels, the abundance of many bacteria were correlated with SBP and DBP. Furthermore, the possible correlation between these shared bacteria and BP levels was also estimated, revealing that among these 26 discriminatory taxa, the abundance of *Rothia mucilaginosa* was positively correlated with host SBP, while a negative correlation between *Thermoanaerobacter indiensis*, *Clostriduim sp. ASBs410* (marginally significant) and DBP were detected (Fig. S5), suggesting they were likely to be explained by differences in SBP and DBP between groups.

### Fecal bacterial function alteration in patients with ISH and IDH

**Fig. 5:**
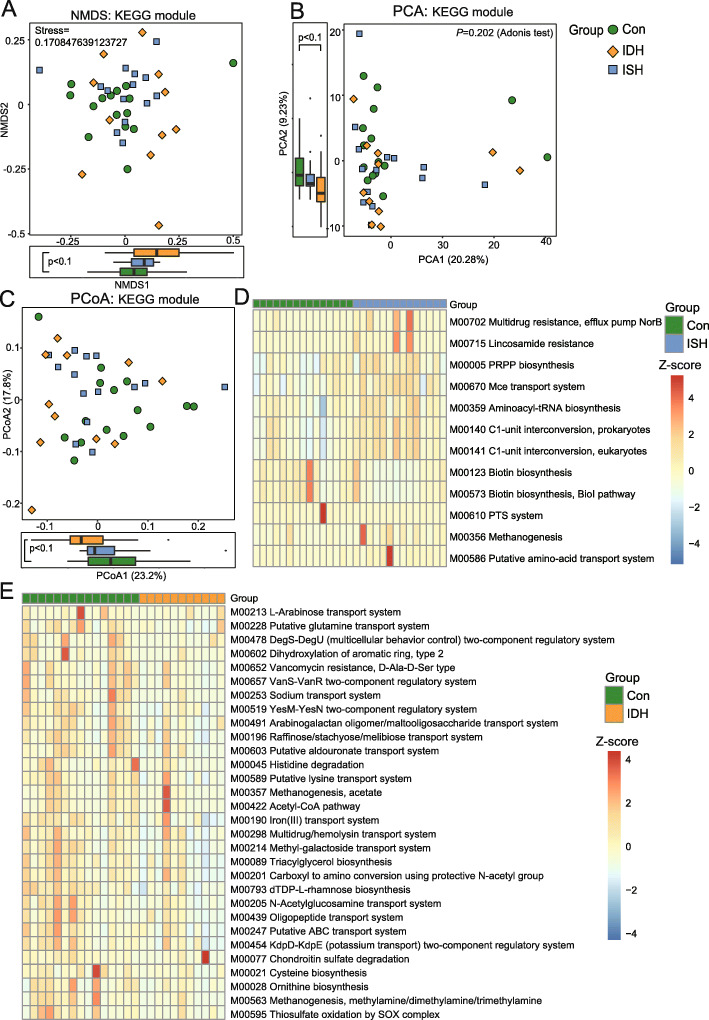
Fecal microbial gene functions were differently enriched between control and IDH, control and ISH. **a**-**c** NMDS, PCA, and PCoA plots based on the relative abundance of KEGG modules in groups. Marginal significant (*P* < 0.1) differences between control (con) and IDH were established at NMDS1, PCA2, and PCoA1; Kruskal-Wallis test. **d** Heat map showing the top 30 differential KEGG modules in control and IDH. The abundance profiles were expressed by Z scores. The Z score was negative (shown in blue) when the row abundance was lower than the mean and is shown in red when the row abundance was higher than the mean. **e** Heat map showing the 12 differential KEGG modules between control and ISH

Using the KEGG database, we assessed the potential gut bacterial functions across groups in the present study (Fig. 5). There was no clear separation in β-diversity between IDH and controls, and alteration in the intestinal bacterial functions in axes was not significant enough (Fig. 5a-c). Again, for the IDH patients, all the significantly altered KEGG modules were decreased in comparison with the control group, such as Sodium transport system, Acetyl-CoA pathway, Triacylglycerol biosynthesis, and Methanogenesis (Fig. 5d). The fecal bacteria function that varied in ISH patients was mainly characterized by nine increased functions, such as multidrug resistance and three reduced functions relevant to Biotin biosynthesis (Fig. 5e).

In addition, the correlation between phenotypic variability of patients including sex, age, SBP, DBP and other demographic characteristics and functionality was assessed by Spearmen’s correlation analysis. We found that several functionality were correlated with SBP and DBP (Fig. S6). Meanwhile, in order to examine the functional redundancy, and the extent to which different species exhibit similar functions, we examined the relationship between species and functional diversity. Spearmen’s rank correlation analysis was performed to examine the relationship between number of species and functions is significant or not, and to identify the number of function increases, decreases or remains stable as species diversity increased [28]. We found strong positive relationships between number of species and number of KEGG modules that functional modules increases linearly with increasing species (*F* = 17.72, d.f. = 61, *P* < 0.001, linear R^2^ = 0.23; Fig. S7).

## Discussion

The extent to which altered fecal bacteria is involved in ISH and IDH remains unclear to the present date. The results of the study have validated our previous hypothesis and provided some novel insights. By performing analysis based on shotgun metagenomic data, the present study discerned the gut bacterial signals in patients with ISH and IDH. Meanwhile, it compared taxonomic composition as well as potential functional modules with healthy controls. We found that compared with controls and ISH, patients with IDH showed a potential decrease in gene number, bacterial richness, and evenness. However, the bacterial alterations did not reach statistical significance in the Shannon index. Differences in the β-diversity were confirmed, and participants with IDH were clearly distinguished from those with ISH at the genus level. More importantly, a few bacterial taxa vanished in IDH, and apparent deficiency of various bacterial genera and species were detected in the taxonomic composition. Patients with IDH were observed to be enriched with *Rothia mucilaginosa*, along with reduced *Clostridium spp.*. Profound bacterial shifts in KEGG functions was noted in patients with IDH, with the sodium transport system and methanogenesis decreased.

The fecal bacteria is known to modify approximately 10% of the host’s transcriptome, especially those related to immunity and metabolism [29, 30], representing the potential signature of HTN. Indeed, a significant decrease in bacterial diversity and an increase of the *Firmicutes/Bacteroidets* ratio were described in spontaneously hypertensive rats [13]. In both pre-hypertensive and hypertensive populations, a dramatic decrease in microbial diversity, *Prevotella*-dominated gut enterotype, and disease-linked functions were also demonstrated [15]. To date, existing studies have compared fecal bacteria features between HTN and healthy controls; however, investigation of fecal bacteria in IDH, ISH, and control populations is still lacking. Even though a previous study [31] reported differences in the abundance of a few genera in the intestinal flora between patients with ISH or IDH with controls, from the independent cohort, as sequenced by 16S ribosomal RNA [31], the potential existence of profound bacterial variance of community structure in ISH and IDH patients has also caused increasing interest. Moreover, a comparison of the similarity and difference of ISH and IDH in bacterial composition and predicted functions of fecal bacteria still need to be further investigated. In the present study, we described the alterations in the intestinal bacteria of ISH and IDH by shotgun metagenomic sequencing data, which further allowed exploration in species-level and function-level.

Bacterial diversity has been regarded as a characteristic related to health status and multiple diseases. Previous studies have frequently reported that when the host is in pathological conditions, fecal bacteria’s diversity is often reduced or significantly altered [32]. Decrease of bacterial richness and diversity have been identified in several chronic diseases, such as HTN [13], Behcet’s disease [33], multiple sclerosis [34], and nonalcoholic fatty liver disease [35] by metagenomic shotgun sequencing, while in the current study, patients with IDH exhibit much deficient genes and a trend toward lower Shannon index, Chao richness and Pielou evenness compared with healthy controls and patients with ISH. Still, some indexes did not reach a statistical significance in the Shannon index. In addition, the β-diversity of participants with IDH differed from controls, as well as from patients with ISH. Based on the aforementioned findings, we concluded that the fecal bacteria structure in IDH patients was more severely perturbed.

Moreover, the variation in taxonomic composition was focused, and specific bacteria were distinct in ISH and IDH. It is noteworthy that the number of genus and species annotated in IDH patients was significantly lower compared to ISH patients, with 50 genera and 200 species completely missing, which is further consistent with reduced α-diversity in IDH. Additionally, compared to healthy controls, we identified that genus *Rothia* and *Rothia mucilaginosa* were enriched in both ISH and IDH. At the same time, *Clostridium spp.* was depleted. *Rothia mucilaginosa*, is a member of the oropharyngeal microbiota and is often detected in the upper respiratory tract [36]. Compelling evidence indicated that certain bacteria can be disseminated from one site to the others and cause systemic diseases [37]. In this regard, numerous studies showed that oral microbes could spread through the body, which have been found in various diseases, such as cardiovascular diseases, adverse pregnancy outcomes, and rheumatoid arthritis [38, 39]. In present study, we found that *Rothia mucilaginosa*, a member of oropharyngeal microbiota often detected in the upper respiratory tract, was enriched in fecal samples from ISH or IDH, which might be due to a possible cavity translocation.

Meanwhile, along with altering the gut bacterial composition, we observed a shifts in bacterial gene functions. Compared with healthy controls, all the KEGG pathways were significantly altered and deficient in IDH patients, such as methanogenesis and sodium transport system. Biosynthesis of methane is believed to have a crucial role in suppressing the inflammatory response and ameliorating oxidative stress injury in various tissues and organs [40–42]. Also, an excessive increase in reactive oxygen production and inflammatory response is implicated in the pathogenesis of HTN [43, 44]. In addition, there is increasing confirmatory evidence that the impaired capacity of sodium transport has an essential role in the development of HTN [45]. Thus, the gastrointestinal dysfunction further verified fecal bacteria’s shifts in IDH and might possibly mediate pathological processes during IDH.

Xie et al. [31] have reported the differences of intestinal flora between ISH patients and controls, and IDH versus controls, respectively by 16S amplicon sequencing. Firstly, the different microbial OTUs in six cases of IDH and six matched cases of normal BP were analyzed. They found five different OTUs between groups, with *Acidaminococcus* and *Megasphaera* higher in IDH, while *Christensenella*, *Lactobacillus*, and *Olsenellathose* higher in individuals with normal BP. Furthermore, the different OTUs in 35 cases of ISH and 35 matched controls were also analyzed. There were 38 different OTUs between groups, such as *Acetobacteroides*, *Aestuariispira*, and *Akkermansia* etc. enriched in ISH patients, *Aeriscardovia*, *Alistipes*, and *Bilophila* etc. abundant in the normal BP group. While in present study, we identified the microbial composition and functional modules among healthy controls, ISH and IDH by metagenomic analysis. We found that compared with controls and ISH, IDH patients exhibited decreased gene number, microbial richness and evenness, although the alterations did not reach statistical significance in Shannon index. The taxonomic composition of ISH or IDH was different from that of healthy controls at both genus and species levels. Patients with IDH or ISH were confirmed to be enriched with *Rothia mucilaginosa*, along with reduced *Clostridium sp. ASBs410*. Lastly, the altered KEGG modules were significantly decreased in IDH compared with the controls, such as sodium transport system; while for ISH, functions relevant to biotin biosynthesis were decreased.

Multiple clinical and observational studies have demonstrated elevated SBP as a more potent predictor of adverse cardiovascular outcomes than DBP in the elderly [46] and emphasized the importance of treating ISH in the elderly so as to reduce the risk of cardiovascular disease events [47]. However, younger subjects should not be ignored as DBP is increasingly predominant and contributes to many adverse outcomes [47, 48]. Khattar et al [48] have compared the prognostic significance of 24-h intra-arterial ambulatory DBP vs. SBP in untreated subjects between aged < 60 and ≥ 60. It was quite interesting that when SBP and DBP were jointly included in the prediction model, DBP (*P* = 0.04) instead of SBP (*P* = 0.67) was related to composite cardiovascular events in the younger subjects. Our findings support a certain extent to previous notions from the aspect of intestinal flora that fecal bacteria of IDH is more disordered in the relatively young subjects.

Our study was the first study to assess the fecal bacteria profiles in participants with ISH or IDH by shoutgun metagenomic sequencing, which allowed to analyze in species-level and functional modules. Meanwhile, there are some limitations. On the one hand, the sample size in our study is relative small. Thus rarefaction curves were performed to evaluate whether the sample size is enough. We found that the curves tend to be flat, indicating that the sample size is sufficient and reasonable, and only a small number of new species characteristics would be produced by more sampling. Moreover, some other studies investigating the gut microbial compositions in diseases, such as type 2 diabetes and obesity, relatively low number of participants with 3 to 6 in each group was also applied [49, 50] Nevertheless, future studies with larger sample size and more patients to validate or check the quantify of these identified species/genus and the findings in the current study by qPCR are still needed. On the other hand, although the reconstructed genomes obtained from the metagenome have the advantage to have longer sequences and cover the entire bacterial 16S rRNA gene for a more detailed taxonomical analysis [12], DNA of dead bacteria could not be excluded from the fecal samples in the whole-genome shotgun sequencing. There is possibility that the DNA in fecal samples detected by DNA-sequencing methods might not be active bacteria. This might hide the real differences among active bacterial members of the different community. As the active bacterial gene expression is affected by gene transcription, metatranscriptomes based methods could be a better indicator of the actual bacteria in future studies [12]. Despite these limitations, the data from present study clearly demonstrate that the fecal bacterial profiles of participants with ISH or IDH.

In conclusion, we described G’s disordered profiles in subjects with IDH and ISH and identified further shifts in IDH. A set of altered bacteria shared between ISH and IDH may comprise features of HTN, which warrants further research. The suppression of specific fecal bacteria, such as translocation of oropharyngeal microbiota, might contribute to pathogenesis. Importantly, our findings also point towards the significance of early intervention for IDH.

## Supplementary Information

**Additional file 1: Table S1.** Data production of fecal samples in control, ISH and IDH.

**Additional file 2: Figure S1.** Rarefaction curves. **(A)** Rarefaction curves by randomly selecting a certain number of individuals from the samples and counting the number of species represented by these individuals in control, ISH and IDH. The curves tend to be flat, indicating the sample size is sufficient and reasonable. **(B)** Rarefaction curves by gradually expanding the sequencing depth of random sampling. The curves approached saturation as the sample sequencing depth increases, and thus the amount of sequencing data is sufficient and stable.

**Additional file 3: Figure S2.** The dispersion of replicates with each group for both bacterial diversity and patient phenotypic variability. (**A)** PCA plots based on the species level in groups. **(B)** PCA plots based on the patient phenotypic variability within each group. A similar with beta-diversity for bacterial community was detected. Instead, individuals in groups vary more in phenotypic variability. Circles in green indicate samples from control, squares in blue indicate samples from ISH, rhombus in orange represent individuals from IDH.

**Additional file 4: Figure S3.** The correlation between phenotypic variability and bacterial diversity by genus level. **(A)** Heat map showing the correlation between phenotypic variability and bacterial diversity at genus level. Indices with |correlation| ≥ 0.2 were shown; Blue, negative correlation; red, positive correlation; *, *P* < 0.05; **, *P* < 0.01.

**Additional file 5: Figure S4.** The correlation between phenotypic variability and bacterial diversity by species level. **(A)** Heat map showing the correlation between phenotypic variability and bacterial diversity at species level. Indices with |correlation| ≥ 0.2 were shown; Blue, negative correlation; red, positive correlation; *, *P* < 0.05; **, *P* < 0.01.

**Additional file 6: Figure S5.** The correlation of shared differential bacteria in ISH and IDH with BP. **(A)** Correlation plots between shared differential bacteria on genus and species level (5 at genus level and 21 at species level) and BP (SBP and DBP). The correlation coefficient is expressed in different colors and sizes. R-value < 0 (negative correlation) are marked with blue circle; r-value > 0 (positive correlation) are in red circle. *P < 0.05, +*P* < 0.1. **(B)** A significant negative correlation between *Thermoanaerobacter indiensis* and DBP (r = − 0.253, *P* = 0.04568) is shown. **(C-F)** Significant correlation between zeta *proteobacterium SCGC AB-604-B04* (r = − 0.255, *P* = 0.04339), *Morganella* (r = − 0.266, *P* = 0.0349), *Rothia* and SBP (r = 0.276, *P* = 0.0286), *Rothia mucilaginosa* (r = 0.283, *P* = 0.0246) and SBP is shown.

**Additional file 7: Figure S6.** The correlation between phenotypic variability and functional modules. **(A)** Heat map showing the correlation between phenotypic variability and functional modules. Indices with |correlation| ≥ 0.2 were shown; Blue, negative correlation; red, positive correlation; *, *P* < 0.05; **, *P* < 0.01.

**Additional file 8: Figure S7.** Functional redundancy was assessed by Spearman’s rank correlation. **(A)** The relationship between number of species and functions by Spearmen’s rank correlation. To examine the functional redundancy, and the extent to which different species exhibit similar functions, we examined the relationship between species and functional diversity by Spearman’s rank correlation analysis. A strong positive relationships between number of species and number of KEGG modules that functional modules increases linearly with increasing species (*F* = 17.72, d.f. = 61, *P* < 0.001, linear R^2^ = 0.23).

## Data Availability

The data set supporting the results of this article is from the EMBL European Nucleotide Archive (ENA) under BioProject accession code PRJEB13870.

## Abbreviations

HTN: Hypertension
ISH: Isolated systolic hypertension
IDH: Isolated diastolic hypertension
SBP: Systolic blood pressure
DBP: Diastolic blood pressure
NMDS: Nonmetric dimensional scaling
PCA: Principal-component analysis
PCoA: Principal coordinate analysis
KEGG: Kyoto Encyclopedia of Genes and Genomes
Nor: Healthy controls
