# Supplementary figures and images for: Characteristics and variation of fecal bacterial communities and functions in isolated systolic and diastolic hypertensive patients
Source: BMC Microbiol. 2021 Apr 26;21:128. doi: 10.1186/s12866-021-02195-1 (PMC8077764; doi:10.1186/s12866-021-02195-1)

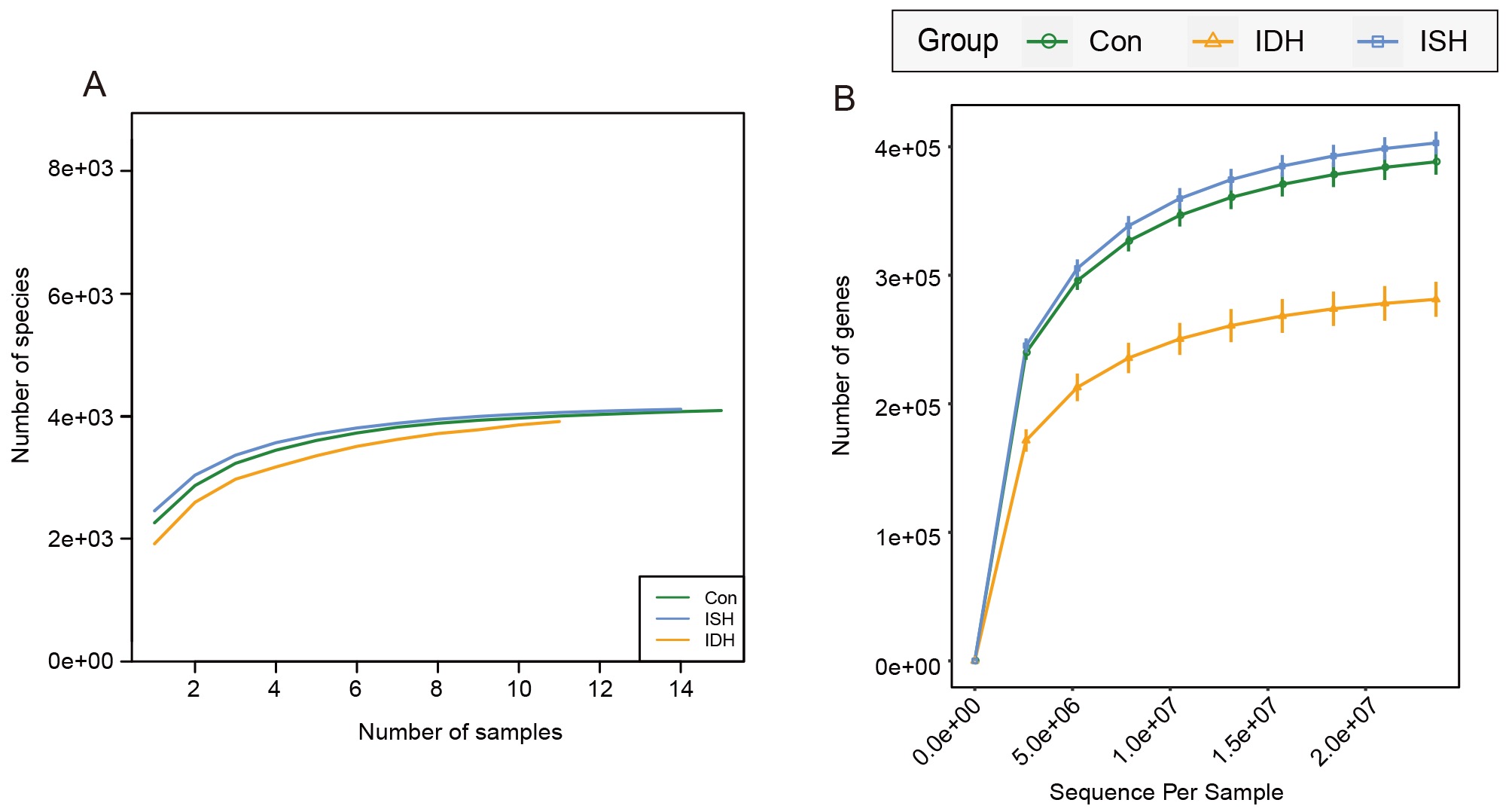

Supplement: Supplementary file 2 — Additional file 2: Figure S1. Rarefaction curves. (A) Rarefaction curves by randomly selecting a certain number of individuals from the samples and counting the number of species represented by these individuals in control, ISH and IDH. The curves tend to be flat, indicating the sample size is sufficient and reasonable. (B) Rarefaction curves by gradually expanding the sequencing depth of random sampling. The curves approached saturation as the sample sequencing depth increases, and thus the amount of sequencing data is sufficient and stable. [file 12866_2021_2195_MOESM2_ESM.jpg]

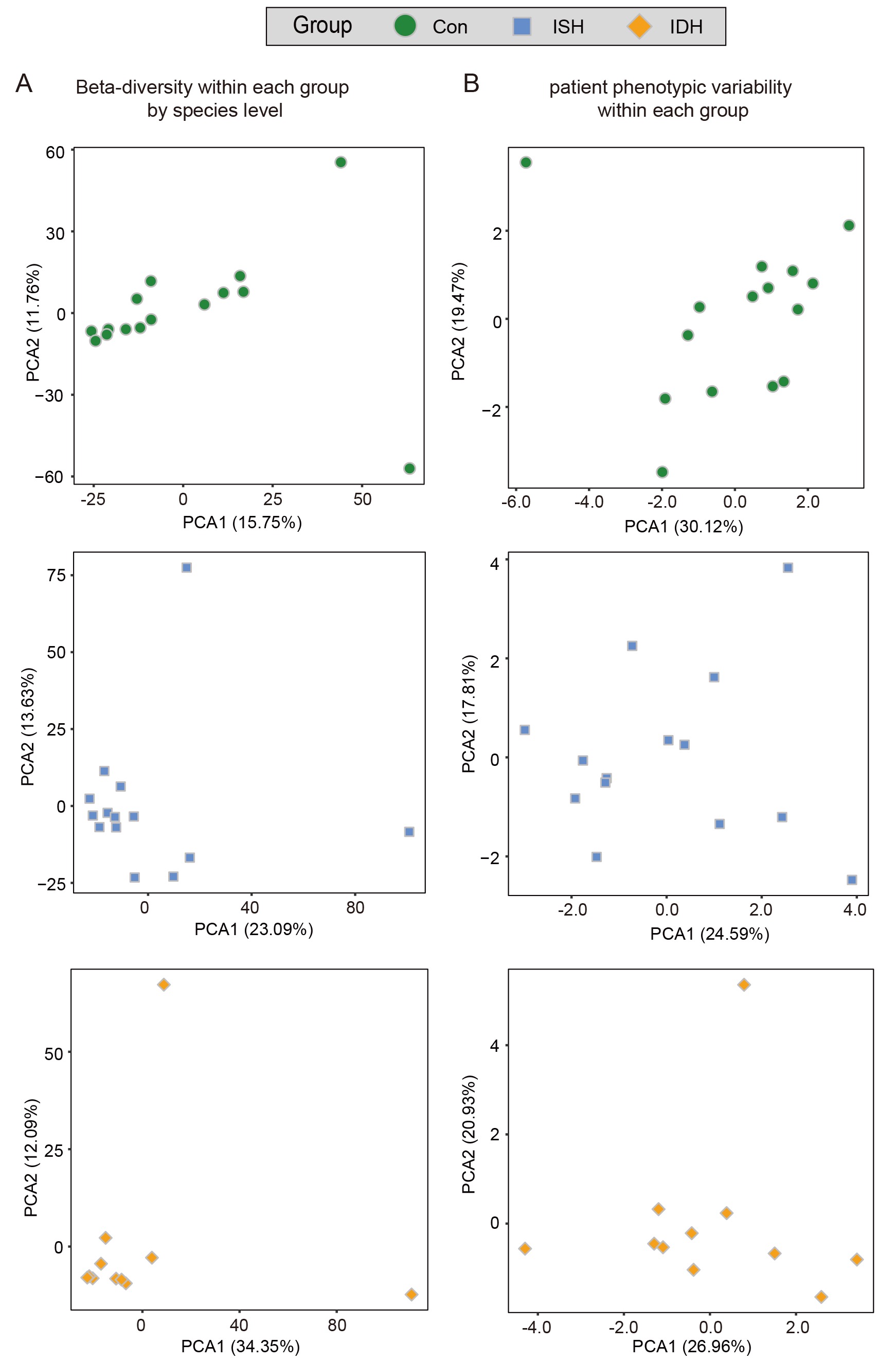

Supplement: Supplementary file 3 — Additional file 3: Figure S2. The dispersion of replicates with each group for both bacterial diversity and patient phenotypic variability. (A) PCA plots based on the species level in groups. (B) PCA plots based on the patient phenotypic variability within each group. A similar with beta-diversity for bacterial community was detected. Instead, individuals in groups vary more in phenotypic variability. Circles in green indicate samples from control, squares in blue indicate samples from ISH, rhombus in orange represent individuals from IDH. [file 12866_2021_2195_MOESM3_ESM.jpg]

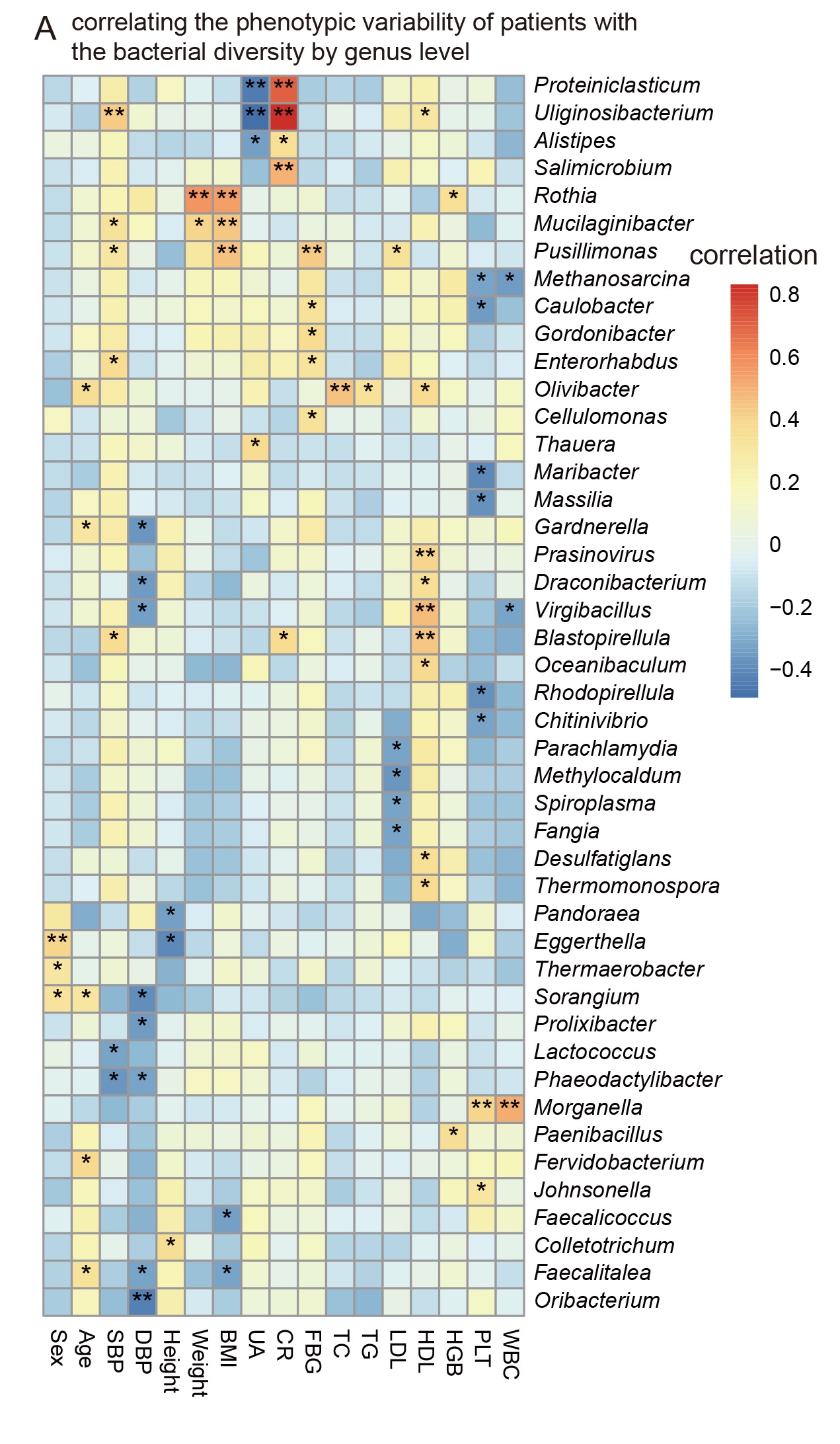

Supplement: Supplementary file 4 — Additional file 4: Figure S3. The correlation between phenotypic variability and bacterial diversity by genus level. (A) Heat map showing the correlation between phenotypic variability and bacterial diversity at genus level. Indices with |correlation| ≥ 0.2 were shown; Blue, negative correlation; red, positive correlation; *, P < 0.05; **, P < 0.01. [file 12866_2021_2195_MOESM4_ESM.jpg]

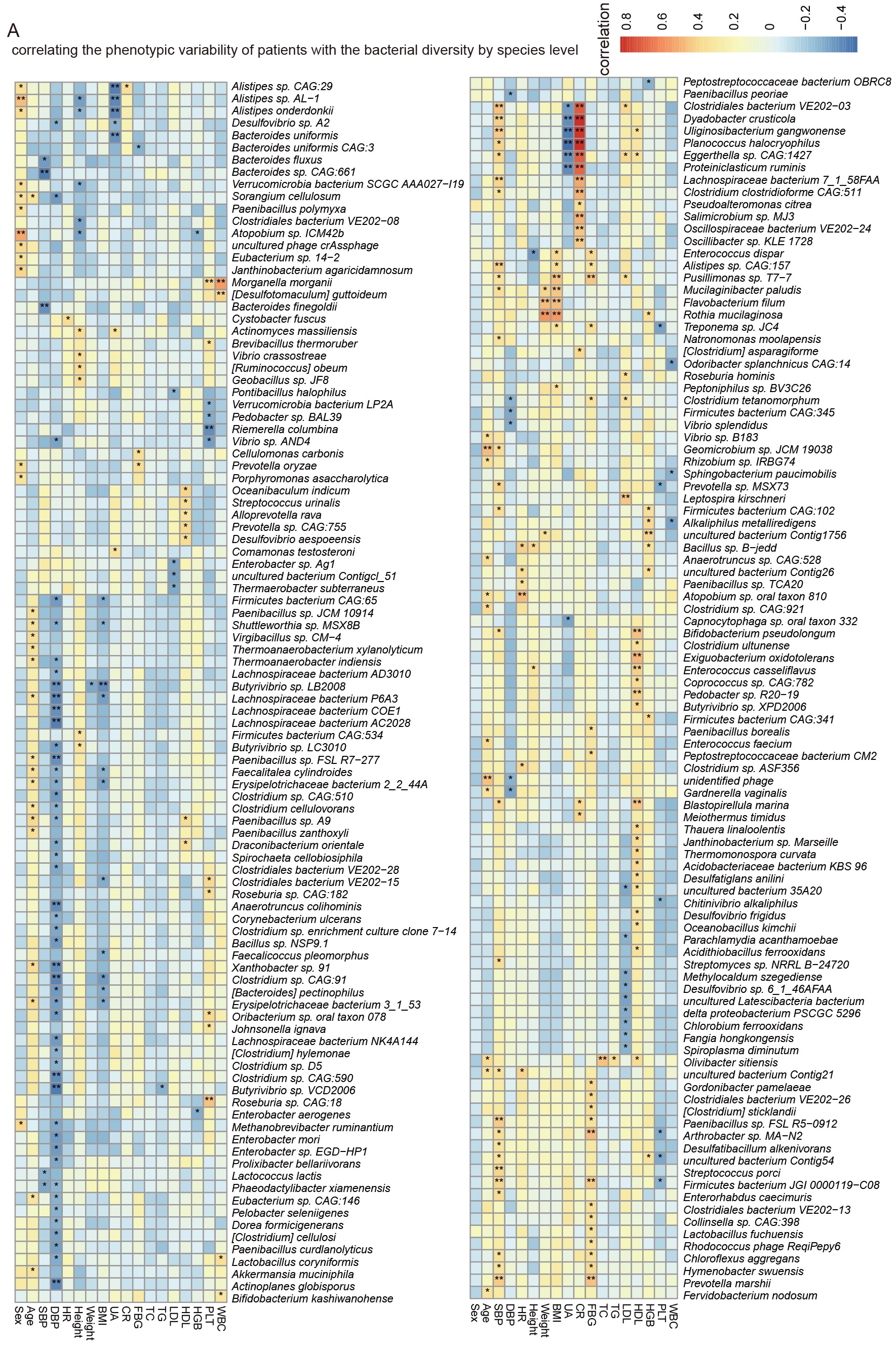

Supplement: Supplementary file 5 — Additional file 5: Figure S4. The correlation between phenotypic variability and bacterial diversity by species level. (A) Heat map showing the correlation between phenotypic variability and bacterial diversity at species level. Indices with |correlation| ≥ 0.2 were shown; Blue, negative correlation; red, positive correlation; *, P < 0.05; **, P < 0.01. [file 12866_2021_2195_MOESM5_ESM.jpg]

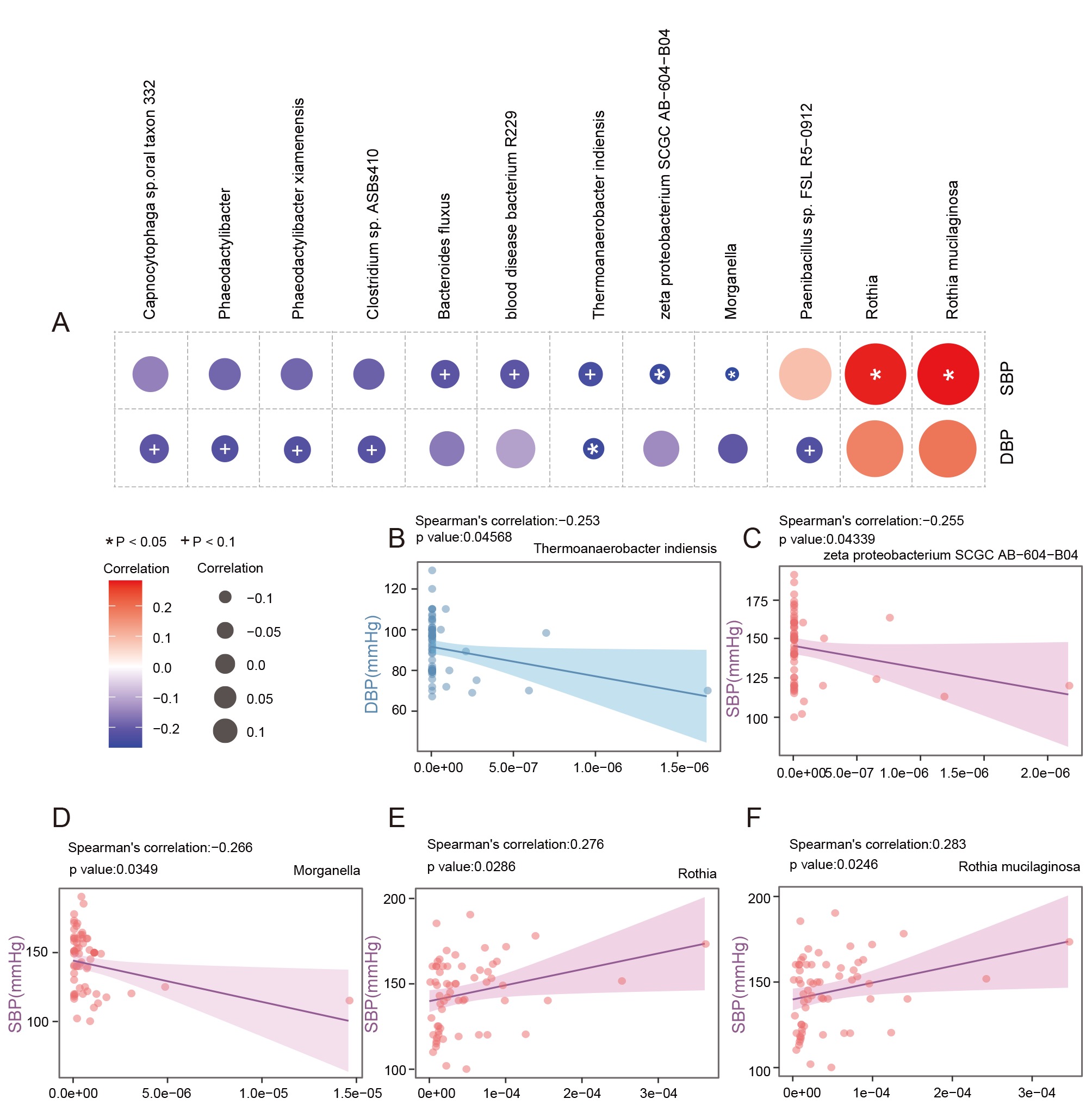

Supplement: Supplementary file 6 — Additional file 6: Figure S5. The correlation of shared differential bacteria in ISH and IDH with BP. (A) Correlation plots between shared differential bacteria on genus and species level (5 at genus level and 21 at species level) and BP (SBP and DBP). The correlation coefficient is expressed in different colors and sizes. R-value < 0 (negative correlation) are marked with blue circle; r-value > 0 (positive correlation) are in red circle. *P < 0.05, +P < 0.1. (B) A significant negative correlation between Thermoanaerobacter indiensis and DBP (r = − 0.253, P = 0.04568) is shown. (C-F) Significant correlation between zeta proteobacterium SCGC AB-604-B04 (r = − 0.255, P = 0.04339), Morganella (r = − 0.266, P = 0.0349), Rothia and SBP (r = 0.276, P = 0.0286), Rothia mucilaginosa (r = 0.283, P = 0.0246) and SBP is shown. [file 12866_2021_2195_MOESM6_ESM.jpg]

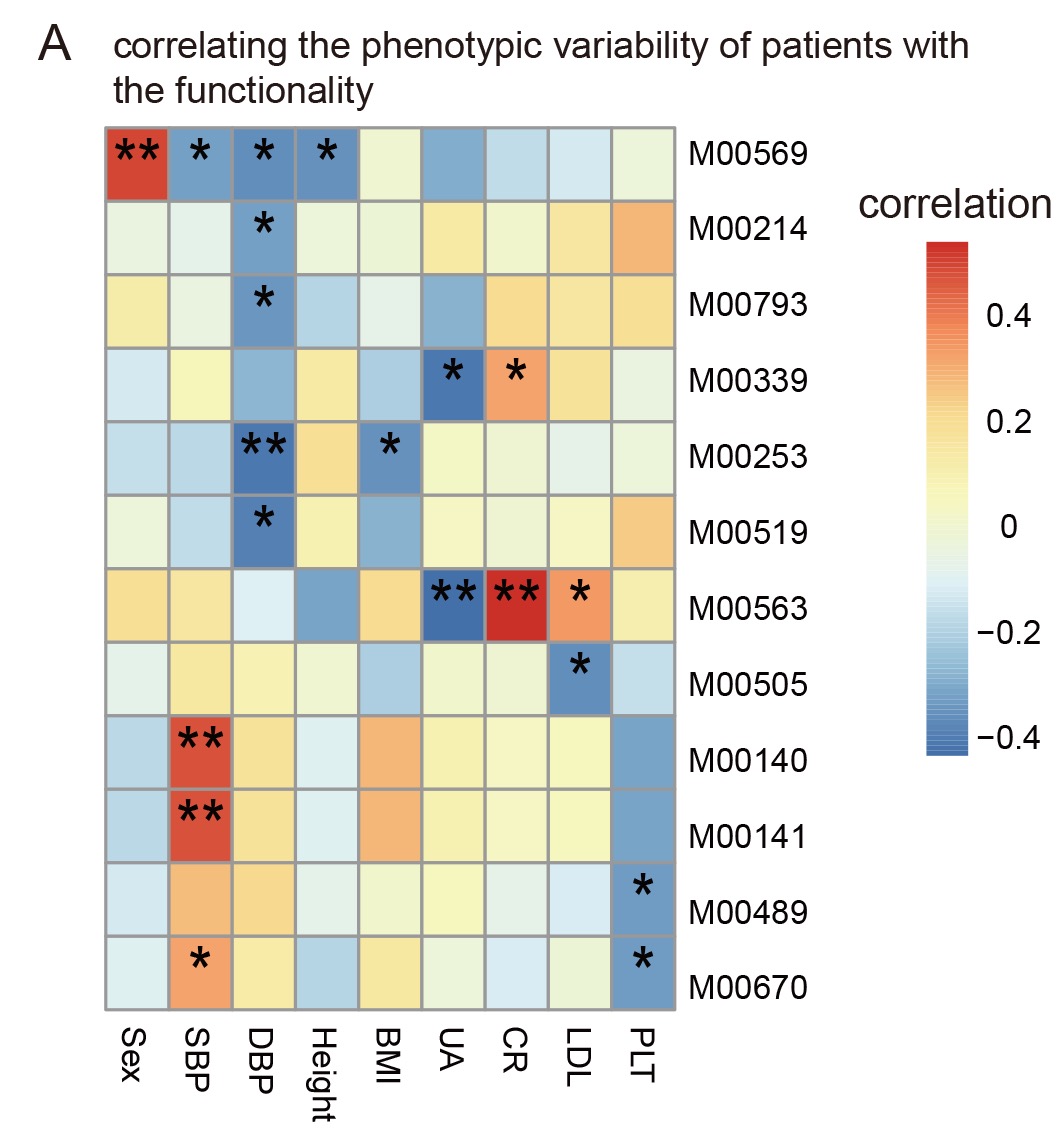

Supplement: Supplementary file 7 — Additional file 7: Figure S6. The correlation between phenotypic variability and functional modules. (A) Heat map showing the correlation between phenotypic variability and functional modules. Indices with |correlation| ≥ 0.2 were shown; Blue, negative correlation; red, positive correlation; *, P < 0.05; **, P < 0.01. [file 12866_2021_2195_MOESM7_ESM.jpg]

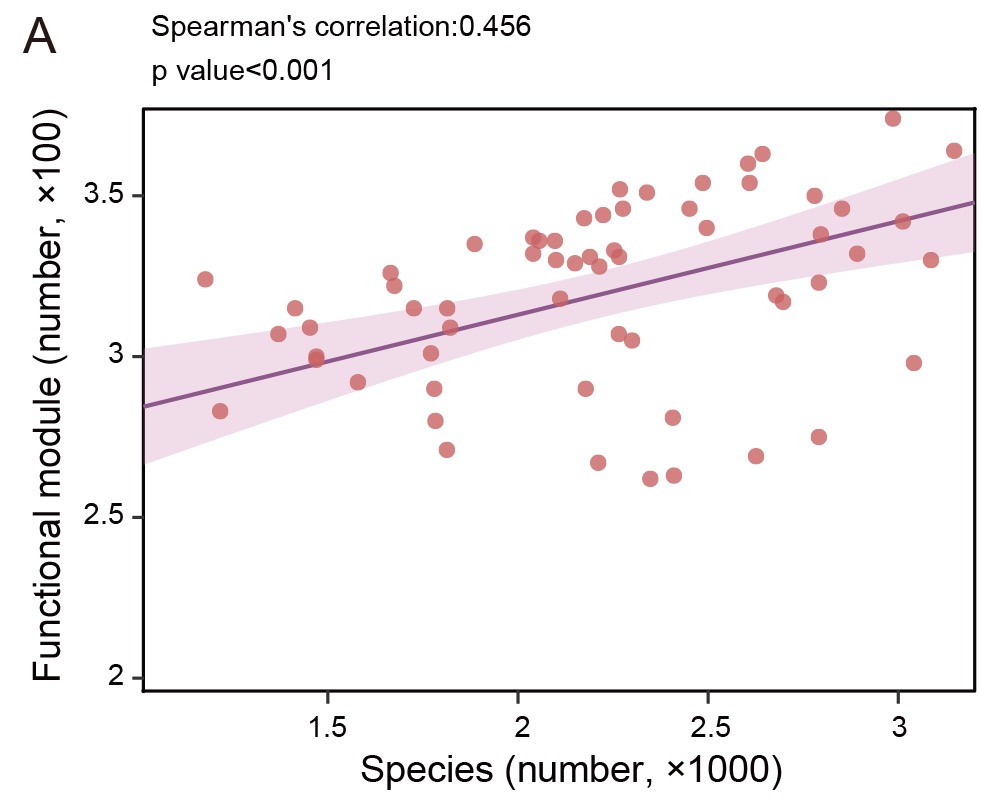

Supplement: Supplementary file 8 — Additional file 8: Figure S7. Functional redundancy was assessed by Spearman’s rank correlation. (A) The relationship between number of species and functions by Spearmen’s rank correlation. To examine the functional redundancy, and the extent to which different species exhibit similar functions, we examined the relationship between species and functional diversity by Spearman’s rank correlation analysis. A strong positive relationships between number of species and number of KEGG modules that functional modules increases linearly with increasing species (F = 17.72, d.f. = 61, P < 0.001, linear R2 = 0.23). [file 12866_2021_2195_MOESM8_ESM.jpg]
